# Child-Directed Speech Facilitates Semantic Role Learning: A Machine Learning Approach

**DOI:** 10.1162/OPMI.a.351

**Published:** 2026-04-17

**Authors:** Eva Huber, Balthasar Bickel, Sabine Stoll

**Affiliations:** Institute for the Interdisciplinary Study of Language Evolution, University of Zurich, Zurich, Switzerland; Department of Linguistics, University of Cologne, Cologne, Germany

**Keywords:** child-directed speech, neural language models, semantic roles, cross-linguistic

## Abstract

Semantic roles, namely the agent (‘doer’) and patient (‘undergoer’) roles, are fundamental to language acquisition, as they enable learners to map meaning onto syntactic structure. Grammars typically impose a binary classification on how these roles map onto syntactic functions. These functions are encoded through features such as agreement, case marking and word order, which children gradually acquire through exposure to their linguistic environment. Notably, child-directed speech constitutes a primary source of linguistic input during acquisition (Hart & Risley, 1995; Weisleder & Fernald, 2013). At present, little is known about the effects of child-directed speech on the learning of semantic roles and even less in languages with diverse grammatical features. Here, we investigate whether child-directed speech facilitates the learning of semantic roles, specifically examining whether it enhances semantic role interpretation compared to adult-directed speech. We examine English and Russian, two languages, which differ fundamentally in how they encode semantic roles, thereby presenting distinct challenges for the language-learning child. We use artificial neural language models to analyse the statistical properties of naturalistic child-directed and adult-directed speech, testing which register more effectively facilitates semantic role learning. In Study 1, we examine whether semantic roles are more easily classified in naturalistic utterances from child-directed speech than adult-directed speech. In Study 2, we test which register better supports learning and generalising semantic roles by evaluating language models trained on either register on the same controlled test set. Study 1 shows that semantic roles are more easily classified in child-directed speech than adult-directed speech, with a more pronounced effect in Russian than in English. This suggests that child-directed speech may be optimised more strongly in a language where semantic roles are expressed in more varied forms and positions, as is the case in Russian. Study 2 shows that the knowledge of semantic roles can be generalised by the models to structures that do not frequently occur in either child-directed speech or adult-directed speech, and that, on the whole, this is more successful based on input from child-directed speech than adult-directed speech in both languages. Our results provide first evidence that child-directed speech is tailored to the language-specific needs of children, facilitating the acquisition of semantic roles, which are a prerequisite for the acquisition of syntax. These findings show that child-directed speech actively supports the acquisition of of semantic roles, helping children map meaning onto syntactic structure.

## INTRODUCTION

A key step in early language development is moving from memorised words and phrases to a flexible, productive system. Semantic roles, that is, the relations between a verbal predicate (e.g., *eat*) and the participants (e.g., *Anne* and *biscuits*), are a critical part of linguistic knowledge. Semantic roles may be specified at varying degrees of granularity (‘eater’ vs. ‘eatee’ or ‘instigator of a causative event’ vs. ‘undergoer of a causative event’). However, it is the fundamental binary opposition between proto-agents (‘doer’) and proto-patients (‘undergoer’) that is systematically mapped onto syntactic functions across languages (Bickel, 2012; Dowty, 1991; Primus, 1999). As foundational components of syntactic rules, binary semantic roles are responsible for morphosyntactic phenomena, such as agreement, case marking and word order (Bickel, 2012).

To identify how languages encode semantic roles, children rely on linguistic input to recognise recurring patterns. This input helps them balance both over- and undergeneralisation as they learn to use language creatively (Behrens, 2020; Lidz & Gagliardi, 2015; Lieven, 2019). Across languages and cultures, child-directed speech (CDS) serves as a crucial source of linguistic input for children’s language development (Golinkoff et al., 2015; Schick et al., 2022; Soderstrom, 2007; The ManyBabies Consortium, 2020). Children’s linguistic competence is strongly associated with the quantity and quality of CDS that they have access to (e.g., Bergelson et al., 2023; Cartmill et al., 2013; Hart & Risley, 1995; Rowe, 2012; Weisleder & Fernald, 2013). CDS encompasses a variety of features that significantly impact language learning. Prosodic features such as elevated pitch and wider pitch range (Fernald et al., 1989; Hilton et al., 2022; Kuhl et al., 1997; Räsänen et al., 2018) capture children’s attention (The ManyBabies Consortium, 2020) and serve a crucial social function in engaging the children in valuable learning opportunities (Golinkoff et al., 2015). These features may further benefit word segmentation (Thiessen et al., 2005; but see Guevara-Rukoz et al., 2018). CDS also has an impact on children’s vocabulary development, shown by the fact that children’s vocabulary size is related to the quantity (Cartmill et al., 2013) of CDS. Additionally, the quality of CDS, in terms of linguistic, conceptual and interactive input features impact children’s language development (Pan et al., 2005; Rowe & Snow, 2020).

We know significantly less about the effect of CDS on the learning of semantic roles and syntax. Certain features of CDS have been identified that may support this stage of language development. First, CDS contains ‘variation sets’—repeated sequences of utterances where the same word is repeated in different morphosyntactic contexts (Küntay & Slobin, 1996; Lester et al., 2022; Wirén et al., 2016). Variation sets expose children to the same lexical root in various morphosyntactic structures, thereby providing cues to its formal and functional properties (Küntay & Slobin, 1996; Lester et al., 2022). Another characteristic of CDS are ‘frequent frames’ (Mintz, 2003; Moran et al., 2018), which are distributional patterns of words in the form of non-adjacent dependencies (e.g., A [] B, the [ADJ] dog). Frequent frames in CDS could facilitate the extraction of word cateogries as words belonging to the same categories occur in similar morphological and syntactic contexts (Lester et al., 2022; Moran et al., 2018; Wirén et al., 2016). Understanding word categories and their morphosyntactic properties is essential for acquiring abstract syntactic rules. Other features of CDS may further assist children in parsing utterances, enabling them to comprehend and generalise rules as they encounter words in different contexts. In particular, utterances in CDS tend to be short (Soderstrom, 2007), contain a low number of clauses (Phillips, 1973), and tend to be lexically restricted in their initial positions (Cameron-Faulkner et al., 2003; Stoll et al., 2009). All of these features considerably facilitate the comprehension of utterances, which, in turn, is crucial for children to follow the conversation, identify how semantic roles are encoded and generalise their knowledge of semantic roles.

In experimental contexts, children correctly interpret semantic roles in frequent constructions (e.g., German (Dittmar et al., 2008), English (Abbot-Smith et al., 2008, 2017) and Korean (Shin, 2021)). In naturalistic CDS, semantic roles can be classified accurately early on in a sentence based on very limited information (Huber et al., 2026). This indicates that in naturalistic interactions, children can understand semantic roles in many if not most instances.

However, it remains unclear whether the characteristics of CDS might still facilitate the extraction and learning of semantic roles or whether the seemingly easy understanding of semantic roles is a characteristic of spoken language in general. A better of understanding of the features of CDS and its impact on learning is crucial for understanding why a higher exposure to CDS is correlated with children’s language proficiency (e.g., Weisleder & Fernald, 2013). We address this question by directly comparing CDS with adult-directed speech (ADS). Concretely, we test whether semantic roles can be more easily extracted and learnt from CDS or ADS. We formalise semantic role learning as a binary classification of agent vs. patient with bivalent (‘transitive’) verbs, and use machine learning methods to implement this task.

Bivalent verbs, i.e., verbs that assign two arguments (e.g., *hit*, *like*, *eat*) describe events involving an agent and a patient. They are regarded as fundamental to human action representation (Gärdenfors, 2014; Hopper & Thompson, 1980) and infant event cognition (Durrant et al., 2021; Leslie & Keeble, 1987; Saxe et al., 2005). Children must learn the binary distinction between these two roles as it accounts for numerous morphosyntactic phenomena across languages and underlies basic syntactic rules (Bickel, 2012). For example, identifying the most agent-like argument is necessary for verb agreement (*-s*) in English (e.g., *The girl eats a hotdog*). Only A (*the girl*) triggers verb agreement (*-s*), while P (*a hotdog*) does not. Therefore, we study semantic roles on the most abstract level, focusing on the binary distinction between the most agent-like and the most patient-like argument of a bivalent verb. These roles have been named ‘proto-roles’ or ‘macro-roles’, indicating their coarse-grained nature (Dowty, 1991) as opposed to more fine-grained roles (e.g., ‘experiencer’ or ‘beneficiary’) or micro-roles (e.g., ‘interviewer’ and the ‘interviewee’ of the verb ‘to interview’). The argument that accumulates most proto-agent properties, such as *volition* or *causing the event*, is considered the agent. Conversely, the argument with the most proto-patient properties, such as *undergoing an event* or being *affected*, is considered the patient. We will refer to these roles as A and P subsequently in order to avoid confusion with the more fine-grained notions of agents and patients of prototypical transitive events, such as *kill* or *hit*. In the proto-role approach, for example, *the cat* is an agent with both a more prototypically transitive verb like *chase* (*The cat chases the mouse*) and perception verbs, like *see* (*The cat sees a bird*).

To assess whether CDS facilitates the learning of semantic roles robustly across different languages, we select two languages, English and Russian, which contrast with each other in how semantic roles are mapped to syntax, specifically in the flexibility of word order and the use of case marking. English has a rigid word order and no case marking except for pronouns. In contrast, Russian allows all six possible word order combinations of A, P and the verb (V), all of which are grammatically correct (Timberlake, 2004). The choice of word order in Russian is influenced by factors such as which part of the sentence is emphasised or which element is the discourse topic (Bailyn, 2012). Russian has a rich declension system and the case of an argument primarily determines semantic roles.

These contrasts pose significantly different challenges for children learning English compared to those learning Russian. Russian-learning children have to cope with a large number of forms of the same lemma (e.g., twelve forms for *sobaka* ‘dog’ as opposed to two forms (plural and singular) for *dog* in English; Gagarina & Voeikova, 2009). In Russian these different forms are contingent on the semantic role of the argument. For instance, in the sentence *koška* (cat.NOM.SG) *gonitsja* (chase.3SG) *za* (for) *myš’ju* (mouse.ACC.SG), ‘the cat chases after the mouse’, the cat in the nominative case is the A that chases after the mouse marked with accusative case, i.e., the P. The meaning remains the same if one changes the word order (e.g., *za myš’ju gonitsja koška*). However, in English, a change in word order alters the meaning of the sentence (*the cat chases after the mouse* vs. *the mouse chases after the cat*), thus word order is decisive for semantic roles in English. In both languages, A-initial sentences represent the canonical word order (Dryer & Haspelmath, 2013). However, Russian-learning children early understand the less frequent P-initial word order by relying on case marking on the arguments (Janssen et al., 2015; Sauermann & Gagarina, 2018). In contrast, English-speaking children often struggle to accurately parse P-initial sentences (Abbot-Smith et al., 2017). Their processing is biased towards interpreting the first noun as an A (Chan et al., 2009), due to the dominance of A-initial sentences in their input (Abbot-Smith et al., 2017).

Similar to children, artificial neural language models (LMs) are excellent statistical learners (Piantadosi, 2024) capable of acquiring sophisticated semantic (Thrush et al., 2020) and syntactic knowledge (Gulordava et al., 2018; Hu et al., 2020). They achieve this by being trained on predicting the next or a masked word, but without being trained on explicit linguistic knowledge. Their neural network architecture further includes constraints on information flow through mechanisms like attention, which are well-suited for the emergence of linguistic structure (Baroni, 2022; Hewitt & Manning, 2019; Manning et al., 2020; Piantadosi, 2024). Notably, You et al. (2021) found that LMs learn word meaning more successfully from CDS than from ADS. Building on this finding, we use LMs to probe whether semantic roles are also more easily learnt from CDS than ADS.

The ease of understanding and learning semantic roles from linguistic input has previously been studied with a cue-based approach (Bates & MacWhinney, 1989; Chan et al., 2009; Garcia & Kidd, 2022; MacWhinney, 1987, 2018). Under this approach, the presence and absence of linguistic cues, such as case or word order, to semantic roles are analysed. Unlike our approach, counting linguistic cues ignores the fact that a learner first has to detect what might count as a cue by analysing distributional patterns in raw linguistic input of the specific language to be learnt. Furthermore, the comparison of registers is restricted to the selected cues, and therefore, likely omits other potential information, such as semantic similarity or contextual information. Thus, unlike earlier descriptive studies on CDS, this approach allows us to directly assess which register provides a more informative source for a statistical learner to identify cues for semantic roles using linguistic input alone. If an LM trained on CDS demonstrates higher accuracy in semantic role classification, we would take this as evidence that CDS plays a key role in facilitating semantic role learning for children. If not, it would suggest that factors like visual and/or prosodic cues outweigh linguistic input in importance for semantic role learning.

For our LMs, we use a scaled-down version of RoBERTa, called *BabyBERTa* (Huebner et al., 2021). BabyBERTa has been shown to learn grammatical features, such as subject-verb agreement and filler-gap dependencies in *wh*-questions. Crucial for our purposes, the model is also susceptible to differences in registers; some structures are learnt better from CDS and others better from written text (Huebner et al., 2021).

We conduct two studies. In Study 1, we investigate whether semantic roles are more easily classified in utterances from CDS than utterances from ADS. In both studies, we train a classifier to predict semantic roles based on argument representations extracted from the trained BabyBERTa model (Huebner et al., 2021). The classification accuracy indicates whether the representations learnt by the BabyBERTa model are more informative with respect to semantic roles based on CDS or ADS. As opposed to cue-based approaches, LMs do not directly make transparent which overt linguistic features (case, order, adpositions, etc.) determine role classification accuracy. In order to further examine the factors influencing role classification accuracy, we test three more types of argument representations that are also usually analysed in cue-based approaches.. First, we analyse to what extent word-level information (i.e., words without context) is sufficient to classify semantic roles. Second, we study the extent to which argument position predicts semantic roles following Connor et al. (2008). Lastly, in Russian, we examine case marking as a predictor for semantic roles. To enhance the interpretation of our results, we supplement this study with a descriptive analysis of word orders, word forms and cases in which semantic roles appear in both CDS and ADS. Word order and case have been frequently studied as cues for semantic roles (e.g., Chan et al., 2009; MacWhinney et al., 1984; Zuban et al., 2021).

In Study 2, we investigate whether CDS benefits learning by comparing classification performance on the same controlled test set between an LM trained on CDS and one trained on ADS. This allows for testing whether a learner exposed to CDS or ADS can more successfully generalise the acquired knowledge of semantic roles to less frequent structures. These include constructions that, according to existing literature, are more challenging for children to acquire, such as patient-initial sentences in English (Abbot-Smith et al., 2017).

The Supplementary Material including data and scripts are available under https://osf.io/4tevz/?view_only=9c5af7e3cd9e493a886808e7c47fc206.

## STUDY 1

### Methods

#### Corpora.

For CDS, we extract adult utterances from longitudinal corpora that track child language development. The CDS data for English is taken from the MPI-EVA-Manchester Corpus (Lieven et al., 2009) and the Manchester Corpus (Theakston et al., 2001). For the Russian CDS dataset, we use the Stoll Corpus (Stoll & Meyer, 2008). The age range of the children for English is 1;8–5;1 (*n* = 16) and for Russian 1;3–6;8 (*n* = 5).

To study ADS, we use the spoken part of the BNC2014 corpus (Love et al., 2017) for English, which consists of transcribed spontaneous conversations among native speakers of British English from across the UK. The conversations were recorded between 2012–2016 and are publicly available. For Russian, we collect the ADS data from two sources. The first part consists of spontaneous conversations between L1 speakers of Russian recorded in 1999 in the Leningrad Oblast (Stoll & Huber, 2023). Since this is a small corpus (37,393 tokens) and since, to our knowledge, no other corpus of spoken Russian is freely available, we resort to open subtitle corpora, parsed and provided by Ebert et al. (2023). Scripted dialogues may differ from spontaneous dialogues in, for instance, the absence of common dysfluencies characteristic of spontaneous speech. For the present purposes, we expect the language in open subtitles to be similar enough in vocabulary and syntax to natural ADS. Due to data limitation, we could not inspect the influence of the open subtitles on the BabyBERTa model itself. However, our sensitivity analyses (see Discussion and S2 in file ‘supplementary_material_additional_analysis’) show that the classification of semantic roles is actually easier in the spontaneous Russian ADS utterances than the ones from the open subtitles.

**Figure 1.**
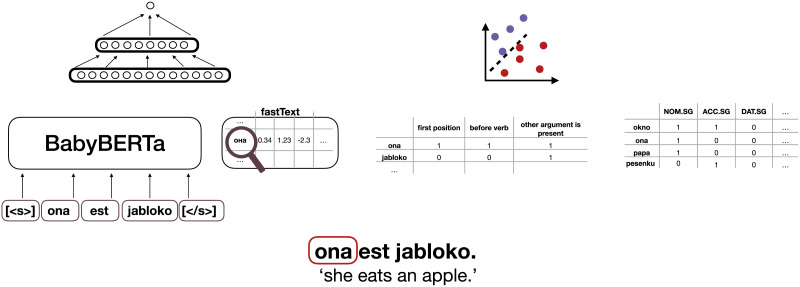
An illustration of the four types of argument representations (B) and the corresponding classifiers (A) for an example utterance (C). For contextualised embeddings (1), we use BabyBERTa (Huebner et al., 2021) and the argument ‘ona’ ‘she.PRO’ is represented in its immediate context. For static embeddings (2), we use word2vec and the argument *ona*’s embedding is looked up from a global vocabulary. For argument position (3), a multi-label vector is annotated with whether the three features (‘first position’, ‘before verb’ and ‘other argument is present’) are true or not. For case (4), a multi-label vector is annotated with the argument word’s forms possible cases. While the form *ona* ‘she.PRO’ occurs exclusively as nominative singular, *okno* ‘window’ is the form of the nominative as well as accusative singular. For study 2, only contextualised embeddings (1) are used.

These data sources serve as the training data for the language models (see 1.B in Figure 1 and 2.1.3) as well as the sources for the representative utterances for both registers that make up our dataset for study 1 (see Annotation of Utterances). We create four training datasets, matched in size for both registers and both languages (approximately 240K tokens). The size is determined by the smallest data set (ADS Russian). For CDS Russian and both English registers, we randomly sampled utterances from their respective datasets once.

We choose not to divide the data by age, but instead use the entire corpus as the training dataset. By doing this, we approximate the quantity of linguistic input that a child has access to as best as possible, but have to disregard the fact that adults adapt their speech to the child’s language development over time (e.g., Tal et al., 2021). In addition, by the time children demonstrate knowledge of the rules that encode semantic roles (e.g., Abbot-Smith et al., 2017) at the age of 2 to 3 years, they likely have had access to more linguistic input than what our models are trained on. For comparison, an average middle-class English-speaking child has access to approximately 10–50M words by about age 6 (Hart & Risley, 1995). However, it is difficult to directly compare the effect of the size of the linguistic input between models and children as the way they are exposed to that input strongly differs. Unlike these models, children learn language in dyadic and group interactions and encounter the input associated to specific activities in bursts over time (Cychosz et al., 2025; Tamis-LeMonda et al., 2019). In addition, the training data for transformer-based models usually are of a larger size including the original BabyBERTa model by Huebner et al. (2021). Due to data constraints of the Russian ADS dataset, we decided to limit all of the training datasets to the same size to ensure comparability across registers and languages. However, future work may use larger training datasets when available to increase statistical robustness.

#### Annotation of Utterances.

We extracted utterances containing bivalent verbs, i.e., verbs with two arguments, from the datasets discussed in the previous section. We follow a semantic definition of valency. Thus, a verb’s valency is not defined based on the syntactic status of its arguments and adjuncts (i.e., whether they are a subject, object or an oblique) but on the argument’s semantic relation to the verb. The valency of a verb is therefore determined by the number of arguments it semantically entails (Dowty, 1991). For instance, the verb *eat* is considered bivalent because the verb entails the presence of someone eating and something being eaten, regardless of the fact that it can be used both with one argument, the A role, (*I’m eating*) and with two arguments, specifying the A and the P (*I’m eating an apple*). Similarly, *go* is considered bivalent since it entails that someone or something moves to a certain goal location (e.g., *The girl* (A) *is going to the park* (P)). Under a syntactic definition of valency, *go* would be a monovalent verb as the goal argument is expressed as an oblique with the preposition *to*. In order to have a list of bivalent verbs, we asked two native speakers of both languages to annotate a list of the most frequently occurring verbs in the datasets with their valency by following our coding guidelines.^1^ In our set of utterances, we only retained those verbs which were annotated as bivalent by both annotators. In the final data set, we only included verbs for which at least ten utterances were available. Therefore, the final number of verbs varies between languages (82 verbs for Russian and 94 for English, see Supplementary Material, S1 in file ‘supplementary_information_study1.pdf’).

We annotated the arguments of the verbs with ‘proto-roles’ (Dowty, 1991), hence we only distinguish between the agent (‘actor’ or ‘doer’) and the patient (‘undergoer’) of an action. The argument of a bivalent verb that accumulates more agent properties (e.g., ‘causing an event’, ‘volitional’, ‘sentient’) is considered the A of an event. The argument accumulating more patient properties (e.g., ‘undergoing an event’, ‘not independently existing’) is considered the P of an event. For instance, in ‘Mary baked a cake’, ‘Mary’ the A argument is volitional and causes the baking event, whereas ‘the cake’, the P argument, does not independently exist and undergoes a change of state.

For English, we used the semantic role parser of AllenNLP (Gardner et al., 2018) to annotate the arguments with semantic roles. This semantic role parser is trained on data annotated by the PropBank annotation scheme which follows Dowty (1991)’s proto-role theory. For Russian, a suitable semantic role parser is currently unavailable. Previous work either employed a different annotation scheme and/or lacks accessible code (Alimova et al., 2020; Kuznetsov, 2015; Larionov et al., 2019). Hence, we used our own pipeline approach. We first parsed the utterances with a dependency parser (UDify parser; Kondratyuk & Straka, 2019). In a next step, we mapped the arguments (nsubj, obj, and obl) to semantic roles using predefined case frames (e.g., *videt’* ‘to see’: <NOM (A), ACC (P)>) from ValPal (Hartmann et al., 2013) and extended by a native speaker of Russian (see ‘data/ru_verbs_and_frames.xlsx’ in Supplementary Material). Utterances in which both arguments (A and P) are omitted were excluded from the dataset. All of the annotated utterances were then manually checked so that the annotations were in accordance with our guidelines of valency and semantic roles. This step, for example, involved removing utterances with monovalent verbs. English has many labile verbs for which both a monovalent (‘The vase broke’) and bivalent (‘I broke the vase’) use exist. We also excluded utterances in which the verbs were used in an idiomatic or highly non-literal way, e.g. ‘to pull funny faces’.

#### Argument Representations.

Our primary focus is on how accurately semantic roles can be classified using the information a statistical learner can derive from CDS and ADS. For this, we use the BabyBERTa model (Huebner et al., 2021), a transformer-based language model (Vaswani et al., 2017) that dynamically assigns a representation to a word based on the context in which it appears (so-called ‘contextualised embeddings’). Contextualised embeddings are particularly well-suited for our study for two reasons. First, they are learnt from statistical distributions in the input, allowing us to assess whether distributions in CDS or ADS allow for better learning of semantic roles. Second, they encode the current word as a function of the surrounding words in the context and can thus encode contextual information, such as word order. Hence, ‘you’ is represented by a different vector in ‘you (A) chase the dog’ as in ‘the dog chases you (P)’. Thus, contextualised embeddings can theoretically bear semantic role information if the input on which the model was trained allows for extracting this knowledge shown by Huebner et al. (2021). However, contextualised embeddings do not give insight into which particular factors are crucial for successful role classification. To better understand this, we test additional representations of arguments in the classification task. Following previous studies on cues to semantic roles (Brandt et al., 2016; Chan et al., 2009; Garcia & Kidd, 2022; MacWhinney et al., 1984), these representations include word-level information, word order and case marking. By examining word-level information, we test to what extent semantic roles can be classified based on the word itself (e.g., ‘you’), without having access to contextual information (‘you’ in ‘you chase the dog’). For representing word-level information, we use ‘static embeddings’, which compared to contextualised embeddings, only assign one representation to each word. For instance, the word *you* is represented by the same vector in both *you* (A) *chase the dog* and in *the dog chases you* (P). Both contextualised and static embeddings are learnt from distributions in the linguistic input. In contrast, for word order and case, we rely on explicit labels. Thus, we provide the classifier with linguistically informed annotations rather than require it to infer this information from latent structures in raw text. We now describe each of the representations in more detail (see B in Figure 1 for an overview of the argument representations).

##### Contextualised Embeddings.

BabyBerta (Huebner et al., 2021) is a scaled-down version of RoBERTa (Liu et al., 2019). BabyBerta differs from RoBERTa primarily in that it has been trained and fine-tuned on CDS, and in that it does not predict unknown tokens.^2^ BabyBERTa has been proposed for smaller training sizes than what larger language models, like RoBERTa, are normally trained on, and is thus ideal for our type of data. We train four language models on each dataset (CDS in English and Russian, and ADS in English and Russian). We use the same hyperparameters as the best performing model in Huebner et al. (2021). The embedding of the argument is retrieved from the whole sentence encoded by the trained language model. RoBERTa, and therefore also BabyBERTa, uses sub-word embeddings to encode the words, resulting in the language model to have access to subword information (e.g., *cheesecake* may be represented as <cheese> + <cake>). However, subwords do not always correspond to meaningful morphemes. Instead, they are frequently occurring character sequences (Gage, 1994; Sennrich et al., 2016) . Subword information may be particularly helpful for a morphologically complex language like Russian. When arguments consisted of multiple words and/or subwords, we averaged their word embeddings to create one embedding.

##### Static Embeddings.

With respect to static embeddings, we use fastText embeddings (Bojanowski et al., 2017). FastText represents words as a bag of characters, unlike other models, such as, ‘Word2Vec’ (Mikolov et al., 2013), which treat words as whole units. We chose fastText embeddings over Word2Vec, because fastText’s use of subword information makes its representations more similar to the contextualised embeddings in BabyBERTa due to the access to subword information. Moreover, fastText is likely a better choice for Russian, given the language’s extensive inventory of word forms and the prevalence of rare words.

We use two types of models to extract static embeddings (2B in Figure 1). First, like for the contextualised embeddings, we train the fasTextmodel on the four datasets (‘static embeddings’ hereafter). The quality of word embeddings is affected by training size. To test to what extent word-level information is sufficient for semantic role classification, without being limited by the limited sizes of our datasets, we also use pretrained fastText embeddings (‘pretrained static embeddings’ hereafter) (Mikolov et al., 2018). These are trained on web-crawled data (number tokens for English: 840B number tokens for Russian: 102B), thus a much higher amount of data than our own trained embeddings.

##### Argument Position.

We represent the argument’s position as a multi-label feature vector (3B in Figure 1). First, the vector encodes whether the argument occurs in first position or not. Second, the vector encodes whether the argument occurs before or after the verb and third, whether the other argument is present. These structural features have been found to be highly predictive of semantic roles (Connor et al., 2008). However, it remains to be seen whether this also holds true for a language with a more flexible word order, such as Russian.

##### Case.

We examine the extent to which case marking on the argument predicts its semantic role (4B in Figure 1). This analysis is only conducted for Russian, as case marking is highly restricted in English, making it a less reliable cue for semantic roles. To encode case, we again use multi-label feature vectors. Each argument is annotated with the possible cases of its surface form. A surface form that serves multiple case functions is considered *syncretic*. In certain declension classes, for instance, the nominative and accusative forms are indentical for inanimate referents, but differ for animate referents (e.g., *krug* ‘circle.SG.NOM’ and *krug* ‘circle.SG.NOM’ vs. *drug* ‘friend.SG.NOM’ vs. *druga* ‘friend.SG.ACC’). By representing the different cases associated with a surface form , we approximate the challenge that a child faces when processing case-syncretic forms and learning the paradigms of each declension class.

#### Classifier.

We implement the task of deciding whether an argument is an A or a P as a binary classification (A in Figure 1). The input is the argument either represented by a contextualised embedding, a static embedding, its position or its case marking. Each data point is one argument as displayed in Table 1. Each argument is classified independently. Thus, in clauses where both arguments are present, the classifier may technically classify both arguments as the same role.

**Table 1.** Examples of data points for the classification task for English and Russian respectively. *A* refers to the agent role, and P refers to the patient role.

Role	Argument	Verb	Utterance
A	he	bite	why did he bite the horse, Anne ? (472754)
P	the horse	bite	why did he bite the horse, Anne ? (472754)
A	*ja* ‘I.PRON’	*kupit’* ‘to buy’	*‘ja xoču kupit’ sok* ‘I want to buy juice’ (J07940922_2421)
P	*sok* ‘juice’	*kupit’* ‘to buy’	*‘ja xoču kupit’ sok* ‘I want to buy juice’ (J07940922_2421)

In case of contextualised and static word embeddings, we train two-layer perceptrons with a hidden layer of size 100. We train the classifier for 20 epochs or until the lowest loss is achieved.

For argument position and case, we use Support Vector Machines (SVMs) with the default non-linear *RBF* kernel, provided by the scikit-learn library (Pedregosa et al., 2011). Initial tests showed that SVMs performed on par with the perceptrons for the feature-based representations, which is why we opted to use the computationally more efficient option for these classifications. The utterances are split into training (100 data points), validation (100–300 data points) and test set (remainder). The training set size was determined by training the classifiers on increasingly larger training sets. With a hundred data points, we strike the ideal balance between achieving high accuracy and ensuring that a sufficient number of data points remain for the test set (see S2 in the Supplementary Material, file ‘supplementary_information_study1.pdf’). Both roles are equally represented in the training set. Verbs that occur in the training set do not occur in the validation or test set, and vice versa, to ensure that the classifier cannot rely on verb-specific patterns. We create ten versions of these splits, each with different verbs represented in each set as we observed fluctuations in performance on the test set depending on the utterances in the training set. We also observed fluctuations in performance across different runs of training the same classifier on the same split. Below we present the results from a randomly chosen run. However, we fitted multiple models on results from different runs and include these results in the Supplementary Materials (Section S1 in the file ‘supplementary_material_additional_analysis.pdf ’). Importantly, the results across runs do not differ from the ones displayed here.

#### Statistical Model.

In the next step, we analyse how register and the particular role (A vs. P) affect the performance of the model. To do so, we estimate the probability of predicting the correct role with Bayesian hierarchical models, using the brms (Bürkner, 2017, 2018) interface to Stan (Carpenter et al., 2017) in R (R Core Team, 2020). The use of statistical models on top of our classifiers allows for directly assessing the weights of these predictors as well as for controlling that the results are not driven by a subset of the sentences in the dataset.

For each language and argument representation of the classifier, we fit one model in order to compare whether there is a difference in performance between the role to be classified and the register of the utterance. As a result, we fit the results across different splits within the same model. We decided to fit separate models for the argument representations instead of a single model because the results across representations are highly collinear, making it impossible to fit a model on all results simultaneously. The outcome variable correct classification is drawn from a Bernoulli distribution with 1 as correct and 0 as incorrect classification. We include register (CDS vs. ADS) and role (A vs. P) and their interaction as main effects and split and sentence id as random intercepts, with random slopes for role.

We choose weakly informative priors for the population-level effects: Normal(*μ* = 0, *σ* = 1) or Normal(*μ* = 0, *σ* = 2), depending on which led to better $\hat{R}$ and effective sample size diagnostics. For group-level effects, we used a weakly informative Exponential(*λ* = 1) prior. The model fit is further evaluated with posterior predictive checks.

For our main analysis, we estimate whether there is a difference between CDS and ADS in the accuracy of predicting semantic roles. To this end, we perform a counterfactual analysis. For each sentence ID, we compute posterior predictions for both registers and calculate the difference between the predictions for CDS and ADS (Δ*P* = *P*(correct classification|CDS) − *P*(correct classification|ADS). We then average the differences across sentence IDs to calculate the overall average difference. A positive average difference indicates that the probability of a correct classification is higher in CDS than ADS, while a negative average suggests the opposite.

### Descriptive Analysis

**Figure 2.**
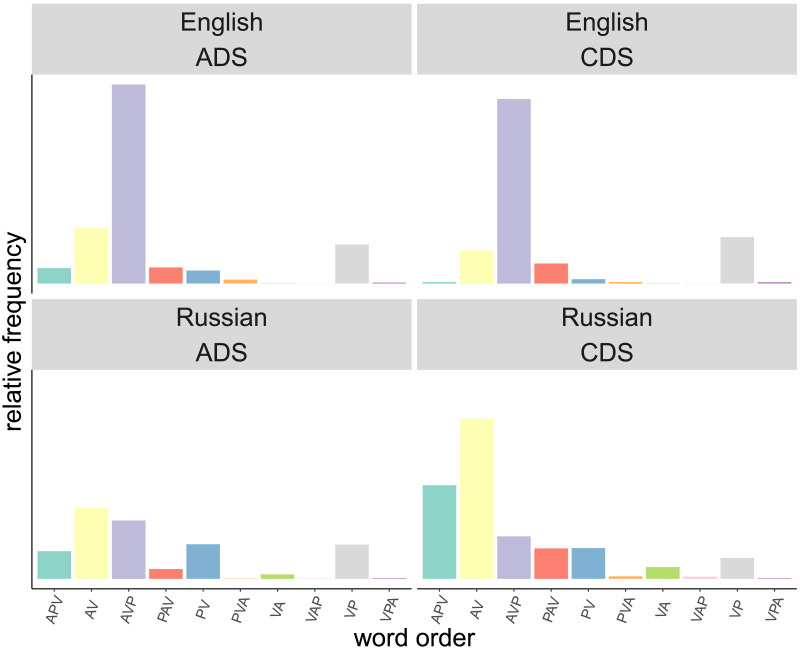
Relative frequencies of word orders in both registers (child-directed speech = CDS and adult-directed speech = ADS) and languages (English and Russian).

We first present a descriptive analysis of the utterances in our dataset to support the discussion of the results. We examine the variability between the two different registers in terms of word order, noun phrase argument and case marking. Specifically, we examine the proportions of word orders and case markings (see Figure 2) as well as their entropy, estimated with maximum likelihood. To calculate the entropy of semantic roles given the argument, word order and case marking (see Table 2) we count how often a given noun phrase functions as A or P. For word order we count the frequency of each word order. Figure 2 shows the distribution of word orders and case marking.

**Table 2.** Entropy values, estimated with maximum likelihood, for argument variability for each role, word order and case for each role (only for Russian).

Type	Language	Role	Entropy CDS	Entropy ADS
argument	English	A	2.24	2.47
argument	English	P	4.86	5.15
argument	Russian	A	3.07	3.86
argument	Russian	P	4.89	5.44
word order	English	A and P	1.15	1.34
word order	Russian	A and P	1.68	1.74
case	Russian	A	0.87	1.80
case	Russian	P	2.03	2.57

A-initial word orders dominate in both registers and languages. As expected, the variation in word order is greater in Russian than in English with more argument omissions in Russian. This is reflected in the entropy values for word order, which is higher in Russian compared to English (1.68/1.74 in Russian vs. 1.15/1.32 in English), indicating greater variability in Russian word order patterns. The distributions of word orders are similar across registers in English with ‘AVP’ (*why did he (A) bite (V) the horse (P), Anne?* (472754 in Lieven et al., 2009)) being the most frequent word order. The second most frequent word orders are ‘AV’ (‘they were cooking’ (743516 in Lieven et al., 2009)) in CDS and ‘VP’ in ADS (*just write (V) it (P) down* (2429 in Theakston et al., 2001)), but the differences in frequency between ‘AV’ and ‘PV’ are small in both registers. Word orders vary more between registers in Russian. In Russian CDS, the most frequent word order is ‘AV’, without a P followed by ‘APV’ (*tak ty paket beri* ‘so you (A) bag (P) take (V)’ (A11640411_1185 in Stoll & Meyer, 2008)). In ADS, the most frequent word order is ‘AVP’ (*A oni edjat jajca?* ‘And they (A) eat (V) eggs (P)?’ (2961_SS in Stoll & Huber, 2023)). Case marking is more varied in Russian ADS than Russian CDS (entropy of 0.87 vs. 1.8 for A and 2.03 vs. 2.57 for P). While many A arguments are nominative singular in CDS, nominative plural is also common in ADS. P arguments are marked by a larger set of cases in ADS than CDS, which results in a higher entropy for case in Russian ADS than CDS. Arguments show greater variability in ADS than in CDS for both semantic roles and languages, as indicated by the higher entropy of arguments in ADS compared to CDS (see ‘argument’ in Table 2).

### Results

Our results show consistently high accuracies of semantic role classification with contextualised embeddings for both languages (Table 3). We find a small, but substantial effect for register, where CDS outperforms ADS for both roles in Russian, but only for the A role in English (Figure 4).

**Table 3.** Study 1: raw accuracies for each language, register, role (A = agent, P = patient) and argument representation. Means and 95% credible intervals of posterior differences between contextualised embedding, a particular role and register and each corresponding combination of argument representation, role and register. For instance, for row ‘static’ and column ‘CDS_A’: *P*_contextualised_(correct classification|register = CDS, role = agent) − *P*_static_(correct classification|register = CDS, role = A). All accuracies are high, clearly exceeding the 0.5 chance level. For English ADS and P, both types of static embeddings show slightly higher accuracies than contextualised embeddings, confirmed by the estimated difference. Argument position for A in both registers in English also shows higher accuracies than contextualised embeddings. In Russian, the static pretrained embeddings outperform contextualised embeddings with P, and case marking representations with all registers and roles, apart from CDS where no difference is found.

Language	Representation	Role	CDS		ADS	
			Acccuracy	Estimated difference	Acccuracy	Estimated difference
English	contextualised	A	0.93		0.88	
English	contextualised	P	0.94		0.94	
English	static	A	0.88	0.034 [0.032, 0.035]	0.87	0.012 [0.011, 0.014]
English	static	P	0.92	0.029 [0.028, 0.031]	0.94	−0.006 [−0.007, −0.005]
English	static pretrained	A	0.86	0.07 [0.069, 0.071]	0.86	0.017 [0.016, 0.019]
English	static pretrained	P	0.91	0.008 [0.007, 0.009]	0.95	−0.008 [−0.008, −0.007]
English	argument position	A	0.99	−0.072 [−0.073, −0.071]	0.97	−0.084 [−0.085, −0.083]
English	argument position	P	0.9	0.049 [0.047, 0.05]	0.84	0.072 [0.071, 0.073]
Russian	contextualised	A	0.9		0.82	
Russian	contextualised	P	0.92		0.9	
Russian	static	A	0.75	0.166 [0.165, 0.168]	0.58	0.228 [0.225, 0.232]
Russian	static	P	0.73	0.185 [0.182, 0.188]	0.69	0.197 [0.194, 0.201]
Russian	static pretrained	A	0.79	0.104 [0.102, 0.106]	0.74	0.078 [0.075, 0.081]
Russian	static pretrained	P	0.95	−0.023 [−0.024, −0.021]	0.88	−0.03 [−0.033, −0.028]
Russian	argument position	A	0.87	0.09 [0.089, 0.092]	0.92	0.003 [0.001, 0.006]
Russian	argument position	P	0.7	0.124 [0.123, 0.126]	0.72	0.087 [0.085, 0.089]
Russian	case	A	0.92	−1.016 [−1.017, −1.014]	0.85	−1.032 [−1.036, −1.028]
Russian	case	P	0.92	−0.996 [−0.998, −0.993]	0.92	−1.032 [−1.035, −1.029]

The accuracy of the classifiers is high throughout with values consistently above the 0.5 chance level (Table 3). Regarding contextualised embeddings, the accuracy values range from 0.82 (Russian ADS, A) to 0.94 (English CDS, P). In English, the highest predictability for A is found with argument position in both registers and for P in CDS with contextualised embeddings. P in ADS is slightly better predicted based on static embeddings than contextualised embeddings (mean of *P*_contextualised_(correct classification|register = ADS, role = P) − *P*_static_(correct classification|register = ADS, role = P) = 0.006, 95% credible interval (CI) = [−0.007, −0.005], see Table 3). In Russian, case marking yields the highest predictability of semantic roles with A (*P*_contextualised_(correct|ADS, A) − *P*_case_(correct|ADS, A) = −1.032, 95% CI = [−1.036, −1.028]).

For the P role, pretrained static embeddings perform best in both register (*P*_contextualised_(correct|CDS, P) − *P*_pretrained static_(correct|CDS, P) = −0.023, 95% CI = [−0.024, −0.021]; *P*_contextualised_(correct|ADS, P) − *P*_pretrained static_(correct|ADS, P) = −0.03, 95% CI = [−0.033, −0.028]). The lowest accuracy values across all results are found with static embeddings in Russian ADS (ADS, A and P roles predicted by static embeddings).

To analyse the effect of register, we calculated the mean posterior difference (Δ*P*) between registers for both roles in each argument representation and language (Figure 4). A positive difference indicates that the probability of a correct classification is higher for CDS than ADS, and vice versa. Values around zero indicate that there is no difference between register.

We observe a positive mean posterior difference of classifying the argument correctly based on contextualised embeddings in English for A (*P*_contextualised_(correct|CDS, A) − *P*_contextualised_(correct|ADS, A) = 0.02, 95% CI = [0.02, 0.03]). However, no effect is found for P, where the credible intervals strongly overlap with 0 (*P*_contextualised_(correct|CDS, P) − *P*_contextualised_(correct|ADS, P) = −0.004, 95% CI = [−.005, −.003]. Thus for English, only A is more easily identified in CDS. In Russian, there is a clear effect for register with both roles (*P*_contextualised_(correct|CDS, A) − *P*_contextualised_(correct|ADS, A) = 0.09, 95% CI = [0.087, 0.092] and *P*_contextualised_(correct|CDS, P) − *P*_contextualised_(correct|ADS, P) = 0.038, 95% CI = [0.035, 0.040]), showing that both roles are more easily classified in CDS than ADS. The effect of register for A is higher than for P in Russian. The effect of register for A is almost five times as high for Russian than for English.

With English static embeddings, we do not find an effect for register with respect to A (*P*_static_(correct|CDS, A) − *P*_static_(correct|ADS, A) = −0.001, 95% CI = [−0.002, −0.001]) and a negative effect for P (*P*_static_(correct|CDS, P) − *P*_static_(correct|ADS, P) = −0.04, 95% CI = [−0.041, −0.039]), i.e., P arguments are more successfully classified in ADS than in CDS. For the pretrained embeddings, we find a negative effect of register for both semantic roles (*P*_pretrained static_(correct|CDS, A) − *P*_pretrained static_(correct|ADS, A) = −.031, 95% CI = [−.032, −.029] and *P*_pretrained static_(correct|CDS, P) − *P*_pretrained static_(correct|ADS, P) = −.02, 95% CI = [−.021, −.019]). In Russian, the results of static embeddings are similar to the ones with contextualised embeddings, i.e., a better predictability of both roles in CDS than in ADS (*P*_static_(correct|CDS, A) − *P*_static_(correct|ADS, A) = 0.149, 95% CI = [0.144, 0.153] and *P*_static_(correct|CDS, P) − *P*_static_(correct|ADS, P) = 0.046, 95% CI = [0.042, 0.05]). The effect of register with pretrained static embeddings is smaller but shows the same trend (*P*_pretrained static_(correct|CDS, A) − *P*_pretrained static_(correct|ADS, A) = 0.057, 95% CI = [0.053, 0.060] and *P*_pretrained static_(correct|CDS, P) − *P*_pretrained static_(correct|ADS, P) = 0.031, 95% CI = [0.029, 0.033]).

Argument position serves as a slightly better predictor for semantic roles in CDS than ADS in English (*P*_word order_(correct|CDS, A) − *P*_word order_(correct|ADS, A) = 0.011, 95% CI = [0.0110, 0.0115] and *P*_word order_(correct|CDS, P) − *P*_word order_(correct|ADS, P) = 0.02, 95% CI = [0.019, 0.022]).

In Russian, we find no difference between CDS and ADS regarding argument position (*P*_word order_(correct|CDS, A) − *P*_word order_(correct|ADS, A) = 0.00, 95% CI = [−.0007, 0.0006] and *P*_word order_(correct|CDS, P) − *P*_word order_(correct|ADS, P) = 0.001, 95% CI = [0.0001, 0.002]).

Finally for Russian, case is a better predictor for semantic roles in CDS than ADS with a larger effect for A (*P*_case_(correct|CDS, A) − *P*_case_(correct|ADS, A) = 0.064, 95% CI = [0.061, 0.068]) than P(*P*_case_(correct|CDS, A) − *P*_case_(correct|ADS, A) = 0.021, 95% CI = [0.019, 0.024]).

### Discussion

In Study 1, we find that contextualised embeddings enable a more accurate classification of semantic roles in CDS than ADS for A in English and for both roles in Russian. This suggests that the statistical properties of the utterances in CDS are better suited to correctly classify semantic roles. Further analysis of additional representations—static embeddings, argument position and case marking—alongside descriptive analysis offers insights into why CDS yields higher predictability for semantic roles.

For English, the higher role probability for A may be driven by a slightly more fixed word order in CDS than ADS. This is reflected in the higher probability of correct classification by argument position in CDS compared to ADS. Whether or not word-level information is also a decisive factor in the difference between CDS and ADS for the A role is unclear. The descriptive analysis and the classification based on static embeddings show conflicting results. The entropy of word forms occurring in arguments is lower in CDS than in ADS for both roles. Nonetheless, classification accuracy is higher in ADS than CDS when using static embeddings (P) and pretrained embeddings (both roles). A possible explanation for this finding may be that the semantic similarity between arguments appearing in a specific role may be higher in ADS than CDS even if the variability in arguments of one role may be lower in CDS than ADS. For instance, an animal may usually be a patient in adult-to-adult conversations (e.g., *I would like a particular type of dog* (1176 in Theakston et al., 2001)), whereas in adult-to-child interactions, animals besides occuring as patients (*you’ve broken that poor piggy now* (626335 in Lieven et al., 2009)) are often also an agent (*Piggy’s having his breakfast* (593981 in Lieven et al., 2009)).

In Russian, the higher performance of CDS than ADS based on contextualised embeddings are likely driven by a lower variability in words and case markers rather than argument position. In the classification results, we find no difference between registers with respect to argument position. Difference of entropy of word order between registers is also negligble (0.06, Table 2), confirming that word order variability remains similar across registers. While a fixed word order could theoretically support children’s early acquisition of semantic roles and case, by providing more reliable cue to semantic roles, this features does not appear to be a specific feature of CDS that supports semantic role learning. The lower variability of arguments in CDS compared to ADS likely contributes to the observed advantage of CDS over ADS. The variability of nouns in arguments in CDS is lower than in ADS (difference of 0.79 in entropy for A and 0.55 for P).

**Figure 3.**
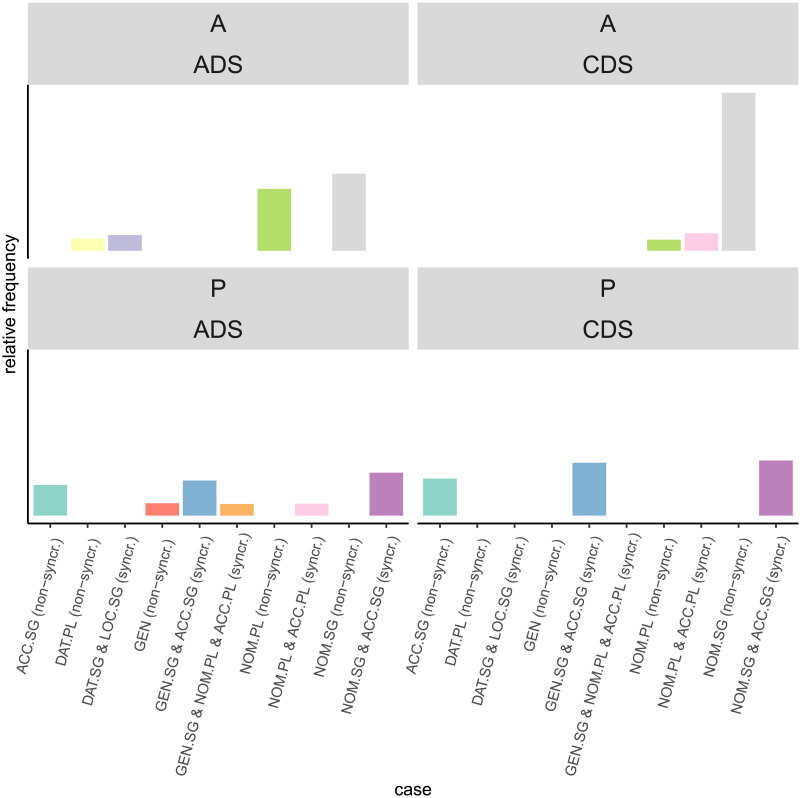
Relative frequencies of case markers in both registers (child-directed speech = CDS and adult-directed speech = ADS) and for both roles (agent = A and patient = P).

Moreover, case marking exhibits greater predictability in CDS compared to ADS. Correspondingly, the variability of case marking is significantly higher in ADS than in CDS, confirmed by an entropy difference of 0.93 for A roles and 0.53 for P roles. The frequencies of different cases in each register (see Figure 3) does not indicate a greater presence of syncretism ADS, but simply a higher diversity in the use of different cases. The lower variability of different cases occurring as the same semantic role may be beneficial for the contextualised embeddings trained on CDS by limiting the number of possible forms used to express a specific role. However, subword units do not necessarily reflect roots and morphemes, and thus the case paradigm may not be easily inferred from the input data. Thus, the precise role of case marking in semantic role classification on the two registers needs to be further examined. Compared to English CDS, Russian CDS may regulate the predictability of semantic roles more based on word forms because of the additional challenge that the morphological system poses to a child learning Russian. Note that since both the entropy calculation and the classification with static embeddings are based on word forms rather than lemmas, the variability of arguments and variability of case marking are not independent measures.

**Figure 4.**
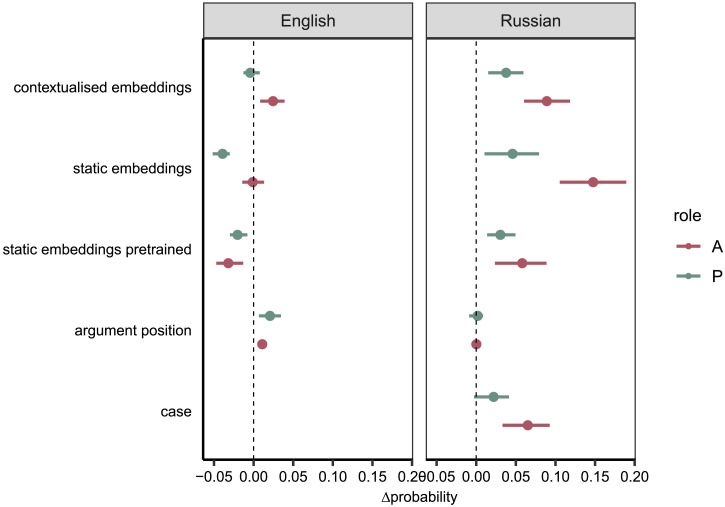
Pair-wise posterior differences between registers (CDS-ADS) for English and Russian on the probability scale, including 95% credible interval, shown for both roles (A = agent, P = patient) and all argument representations. In English, the probability of a correct classification is only higher in CDS with argument position and for A also with contextualised embeddings, with Δ*P* > 0. With contextualised embeddings for P roles and static embeddings for A roles, no difference between CDS and ADS is detected, while for static embeddings P and pretrained static embeddings, Δ*P* < 0, meaning that the probability of classifying the role correctly is higher for ADS. In Russian, the Δ*P* is consistently above 0 for all argument representations and both roles in Russian, except for argument position.

Prepositions may be a further indicator for semantic roles with verbs whose arguments are headed by a preposition (e.g., the P argument of ‘go’ is headed by the preposition ‘to’ in English and ‘na’ in Russian). While we do not investigate them as argument representations seperately, they could serve as an additional cue represented in the contextualised embeddings and, thus, used by both the BabyBERTa model and children to determine the role of an argument.

Importantly, contextualised embeddings capture the semantic role learning best, while the other representations serve to better understand these results. It is to be expected that some of the other representations achieve higher accuracies than contextualised embeddings. First, word order and case marking represent human-annotated labels, whereas a child, similar to the BabyBERTa model, has to learn such features from statistical distributions in the input. Second, pretrained embeddings were trained on a substantially larger amount of data, enhancing the quality of their word embeddings. Nevertheless, we note that static embeddings trained on our datasets also performed well in some cases. We take the strong performance of pretrained embeddings, and in some cases, static embeddings, as evidence that word-level information is often sufficient for semantic role classification. In other words, many words are strongly associated with one of the two roles.

Interestingly, the effect size of the difference in register with contextualised embeddings is higher in Russian than English. This shows that there is a stronger positive effect of CDS for semantic role learning in Russian than in English.^3^ The difference in effect size can be explained by the language’s syntactic differences. Given that word order serves as a more reliable cue for English-learning children than for Russian-learning children, and that English largely lacks case, identifying ‘who does what to whom’ may inherently be easier in English than in Russian, regardless of register. This is confirmed by the accuracy scores which are higher throughout in English than in Russian (Table 3). Hence, there may be less room for a particular register to facilitate the learning of semantic roles when these roles are highly predictable from the outset, as in English, compared to Russian, where they are less predictable. Moreover, if semantic roles can easily be learnt, there is less pressure for CDS to adapt its structures to facilitate communication and increase learnability.

## STUDY 2

In Study 1, we found that a limited set of word orders are dominant in both registers and that argument position is a reliable predictor for A in both languages. This raises the question of how well a learner can generalise beyond these frequent structures. It remains open whether semantic roles can simply be more easily classified in CDS because of the nature of the utterances in CDS or whether they can also be more easily *learnt*, which requires generalising knowledge to various structures. In Study 2, we now test which register provides a better source for learning semantic roles. For this, we evaluate the LMs trained on either register on the same controlled test set in which we manipulate different features relevant for semantic role classification. Here, we are interested in understanding to what extent a learner acquires knowledge of semantic roles based on the statistical properties of words and constructions in a particular register. Thus, we only use contextualised embeddings as argument representations.

### Methods

#### Controlled Dataset.

Our controlled datasets comprise both sentences reported as difficult to acquire and those regarded as less challenging. To construct them, we manipulated sentences according to several conditions. In the English dataset, we manipulate the prototypicality of referents and word order (Table 4). In Conditions 1 and 2, the sentences follow the most frequent order: ‘AVP’. In Condition 1, A is human and P is animate (animal) or inanimate, corresponding to the most prototypical bivalent event, i.e., a human acting (Hopper & Thompson, 1980). We call this the *base* condition. Semantic roles of non-prototypical referents are more difficult for children to interpret correctly (Corrigan, 1988). Thus, in Condition 2, we flip the type of referents, i.e., P is human and A is an animal or inanimate. Here, the model needs to rely on word order since the sentences are sometimes implausible or metaphorical (e.g., *The stations come to you*), and the referents do not correspond to the most typical As or Ps. In Conditions 3 through 5, we manipulate the word order to test whether the model correctly classifies arguments in utterances with the less frequent P-initial word orders ‘PAV’ and ‘PVA’. In a number of studies, P-initial sentences have been found difficult for English-speaking children to acquire (e.g., Abbot-Smith et al., 2017; Armon-Lotem et al., 2016; Macdonald et al., 2020). We create ‘PAV’ sentences by fronting P in Condition 3, relativising P in Condition 4 and ‘PVA’ sentences by putting the sentence into passive voice in Condition 5. By including structures with infrequent word orders, we test to what extent the model relies on the most frequent word order (AVP) or has had sufficiently informative input to generalise the knowledge of semantic roles to less frequent constructions.

**Table 4.** Conditions and examples for the English controlled dataset.

**Condition**	**Example**
Basic	*The ladies sit on the chairs.*
Unprototypical animacy	*The car runs to the girls.*
Fronted	*The toys, the baby wins.*
Relative clause	*The houses that the person hides behind to are white.*
Passive	*The lid is thrown by the monkeys.*

In all conditions, we include sentences with either the A argument or the P argument as pronouns besides lexical NPs. In spoken language, transitive verbs occurring with two lexical arguments are rare (Haig & Schnell, 2016), which may pose an additional challenge to the classifier and results in the classifier having to solely rely on grammatical information. For pronominal arguments, we use the third person singular (*she*/*her*) and plural (*they*/*them*) which have distinct forms for nominative and accusative/dative case as well as the second person singular/plural (*you*) which only has one form and is therefore more ambiguous with regard to its semantic role. Lastly, we manipulate number (singular vs. plural) so that in half of the sentences agreement is available as a cue (e.g., *To the ships, the boy goes.*) and in half of the sentences it is not (resp. *To the ships, the boys go.*).

In the Russian data, we manipulate both case syncretism and word order (Table 5). Syncretic forms, i.e., nouns where different cases share the same surface form, make the case system less reliable and therefore can be dificult for children to acquire (Gagarina & Voeikova, 2009; Peters, 1997). Children’s strategies for classifying semantic roles shift from depending on word order to recognising and using case markers (Akhutina et al., 2019; Janssen et al., 2015; Sauermann & Gagarina, 2018). Thus, studying different word orders gives insight into whether a learner relies on either case marking or word order. If the contextualised embeddings capture information about case marking, we expect non-syncretic forms to be not only classified better in frequent word orders, where both word order and case marking cues are present, but also in less frequent word orders, where the cue of word order is absent. Alternatively, the classifier may simply rely on word order which would be shown by a similar accuracy across syncretic and non-syncretic forms. In Condition 1, case markings of neither A or P arguments are syncretic, i.e., we choose nouns of declension classes that assign distinct cases to nominative (e.g., *čelovek* ‘NOM.SG.person’ ) and accusative (e.g., *čeloveka* ‘ACC.SG.person’). In Condition 2, both arguments are assigned syncretic cases (e.g., *mat’* ‘NOM/ACC.SG.mother’). Lastly, we include two conditions where either the A argument (condition 3) or the P argument (condition 4) is syncretic. Conditions 1 through 4 are included in the dataset with all possible word orders, which is any order of the set {A, V, P} in Russian. As with English, we also manipulate the number of the argument so that agreement is available or not available as a cue. For Condition 2, however, we only include sentences where agreement is a cue since without it, the sentence would be ambiguous with regard to its semantic roles (e.g., *mat’* ‘NOM/ACC.SG.mother’ *lovit* catch.3SG *doč’* ‘NOM/ACC.SG.daughter’ could mean ‘the mother catches the daughter’ or ‘the daughter catches the mother’).

**Table 5.** Conditions and examples for the Russian controlled dataset.

**ID**	**Condition**	**Example**
1	non-syncretic	*dočka ljubit sobak* ‘NOM.SG.daughter 3SG.love ACC.PL.dog’
2	both arguments syncretic	*mat’ kormit dočerej’* ‘NOM.SG.mother 3SG.feed ACC.PL.daughter’
3	syncretic A	*myš’ idet v komnatu* ‘NOM/ACC.SG.mouse 3SG.go to ACC.SG.room
4	syncretic P	*mama deržit hleb* ‘NOM.SG.mommy 3SG.hold NOM/ACC.SG.bred’

For both datasets, the sentences are generated using a semi-automatic process. To do so, we choose a list of nouns and verbs that are represented well in the corpora (*N* > 10). We try to choose a balanced set of representative nouns of both registers. We manually add constraints on how different verbs and nouns can be paired, and the full list of sentences is then generated automatically. The goal is not to create a naturalistic set of sentences as they would occur in everyday language, but rather to design a controlled set that tests the limits of the language models’ grammatical knowledge. The final dataset consists of 21,600 sentences for English and 73,884 sentences for Russian.

#### Argument Representations.

Study 1 controlled for the training data to be similar in size across languages. We do not compare the results across languages in Study 2 as the structures in the controlled dataset are language-specific. Thus, for English, we train a new English BabyBERTa on the full dataset (see Corpora) as well as a sizematched subsample of the ADS so that the datasets match in size between register. We use the same BabyBERTa models for Russian as in Study 1 (see Contextualised Embeddings in Argument Representations) as it was already trained on the full datasets at hand.

#### Classifier.

We train the same architecture as in Study 1 (Classifier section) for our classifiers. We split the controlled data sets into training (100 sentences), validation (200 sentences) and test (remainder) sets. In the test sets, we attempt to represent each condition in accordance with how often they would occur in natural language. For English, the ‘unprototypical animacy’ condition and the PVA conditions (‘fronted’, ‘relative clause’ and ‘passive’) occur four times (two times for each role). The rest of the dataset constitutes sentences in the ‘basic’ condition. For Russian, we represent the conditions sentences in proportion to how often each word order occurs in CDS (APV: 52, AVP: 24, PAV: 18, PVA: 2, VAP: 2, VPA: 2). The syncretism conditions are sampled randomly. For both English and Russian, each condition occurs at least once in the classifier.

#### Statistical Model.

We fit the same type of model and priors as in Study 1 described in Statistical Model. We again include register and role as main effects. In addition, in the case of English, we fit a main effect for sentence manipulation for English, condition. For Russian, we add a main effect of whether the argument is syncretic or not, syncretism and an additional main effect of the word order of the sentence, word order. We additionally include a random intercept for sentence ID and let it vary by register. As in Study 1, we use global, weakly regularising Normal(*μ* = 0, *σ* = 1) priors for the population-level and Exponential(*λ* = 1) priors for group-level effects.

### Results

The results show that in both languages, semantic roles are on the whole more accurately classified based on the BabyBERTa models trained on CDS than the ones trained on ADS. We observe some variation among conditions.

In English, the accuracy is highest with sentences in the canonical AVP word order (see Table 6). The manipulation of the referent prototypicality does not impact the predictability of semantic roles with both ‘prototypical’ and ‘unprototypical’ animacy achieving high accuracy levels. Relative clauses are more easily classified than fronted clauses with regard to both roles and registers. Passive sentences are classified least well. In both registers, A is classified below chance, i.e., misclassified as P. The P argument is classified above chance by both registers.

**Table 6.** Study 2 English: raw accuracies for each register, role (A = agent, P = patient) and condition. Means and 95% credible interval of posterior differences between ‘plausible animacy’, a particular role and register and each corresponding combination of condition, role and register. For instance, for row ‘fronted’ and column ‘CDS_A’: *P*_plausible animacy_(correct classification|register = CDS, role = agent) − *P*_fronted_(correct classification|register = CDS, role = agent). All accuracies are high, clearly exceeding 0.5 chance level, apart from A in the ‘passive’ condition. Both roles in the conditions ‘plausible animacy’ and ‘implausible animacy’ (AVP word order) are classified most accurately, followed by relative clauses which are in turn followed by fronted clauses. In the ‘passive’ condition, only P is classified above chance.

Condition	Role	CDS		ADS	
		Accuracy	Estimated difference	Accuracy	Estimated difference
plausible animacy	A	1		1	
plausible animacy	P	0.97		0.97	
implausible animacy	A	1	0.0017 [0.0016, 0.0019]	1	0.0027 [0.0025, 0.0029]
implausible animacy	P	0.94	0.0098 [0.0092, 0.0104]	0.92	0.0073 [0.0068, 0.0077]
fronted	A	0.81	0.19 [0.1851, 0.1883]	0.86	0.16 [0.155, 0.158]
fronted	P	0.86	0.09 [0.0981, 0.1013]	0.77	0.19 [0.1857, 0.1892]
relative clause	A	0.98	0.016 [0.0154, 0.0165]	0.94	0.07 [0.0642, 0.0662]
relative clause	P	0.91	0.05 [0.047, 0.049]	0.9	0.09 [0.084, 0.087]
passive	A	0.43	0.518 [0.516, 0.5199]	0.33	0.6579 [0.6558, 0.66]
passive	P	0.77	0.192 [0.190, 0.194]	0.72	0.246 [0.24, 0.25]

**Figure 5.**
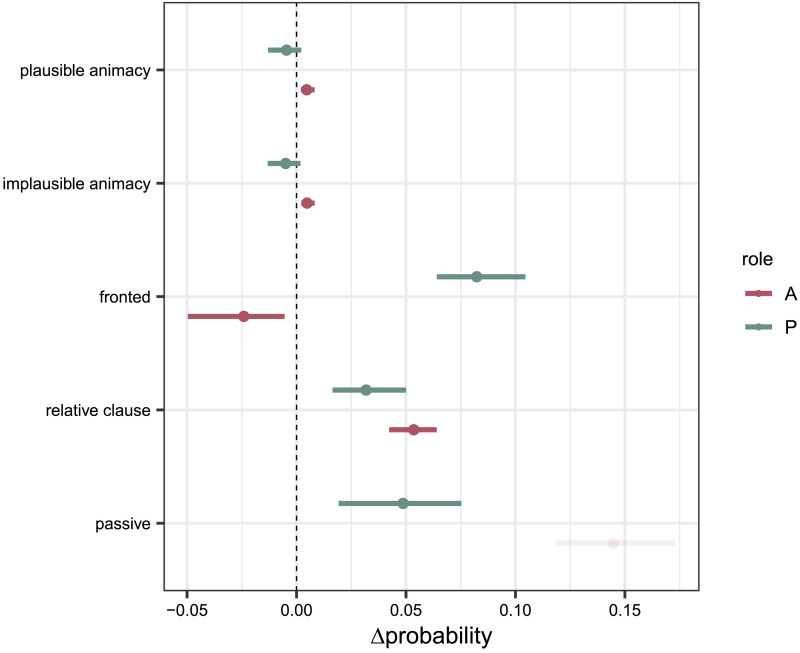
Pair-wise posterior differences between registers (CDS-ADS) for the English controlled dataset on the probability scale, (Δprobability), including 95% credible interval, shown for both roles (A = agent, P = patient) and all conditions. Regarding the ‘plausible’ and ‘implausible animacy’ conditions (word order AVP), we find a slightly higher predictability for the CDS LM in the case of A, and a slightly higher predictability for the ADS LM in the case of P.

Figure 5 shows that for English there is only a slight difference between CDS and ADS for sentences in the canonical word order with a small benefit of CDS for A (*P*_plausible animacy_(correct|CDS, A) − *P*_plausible animacy_(correct|ADS, A) = 0.005, 95% credible interval (CI) = [0.0047, 0.0054]) and and a slim benefit of ADS for P (*P*_plausible animacy_(correct|CDS, P) − *P*_plausible animacy_(correct|ADS, P) = −0.005, [−0.006, −0.004]). Regarding the ‘fronted’ condition, predictability of CDS is higher for P (*P*_fronted_(correct|CDS, P) − *P*_fronted_(correct|ADS, P) = 0.08, [0.081, 0.09]) but not for A (*P*_fronted_(correct|CDS, A) − *P*_fronted_(correct|ADS, A) = −0.025, [−0.028, −0.023]). With the ‘relative clause’ condition, however, CDS yields higher predictability than ADS with both roles (*P*_relative clause_(correct|CDS, A) − *P*_relative clause_(correct|ADS, A) = 0.054, [0.053, 0.055] and *P*_relative clause_(correct|CDS, P) − *P*_relative clause_(correct|ADS, P) = 0.033, [0.031, 0.034]). The same is true for the ‘passive’ condition with P (*P*_passive_(correct|CDS, P) − *P*_passive_(correct|ADS, P) = 0.049, [0.046, 0.051]). In both registers, A is classified below chance, thus we do not further discuss the effects of register here.

The results in Russian vary highly across different word order and syncretism conditions (see Table 7). The highest accuracy values, consistent across roles, are found with the most frequent word orders ‘AVP’, ‘APV’ and ‘VAP’, for both registers. We do not find large differences in predictability across the different syncretism conditions of the same word order. Figure 7 shows the estimated probability of a correct classification for each syncretism condition and semantic role grouped by word order and register. The estimated probabilities across syncretism conditions are similar when looking at the same semantic role, register and word order, indicating that non-syncretic arguments do not tend to be classified better than syncretic ones. Marginalising the effect of syncretism even shows that the ‘both syncretic’ leads to the highest estimated probability of a correct classification (‘non-syncretic’: mean = 0.69, 95% CI = [0.69, 0.69], ‘syncretic A’: 0.78 [0.77, 0.78], ‘syncretic P’: 0.70 [0.70, 0.70] and ‘both arguments syncretic’: 0.82 [0.82,0.82]).

**Table 7.** Study 2 Russian: raw accuracies averaged over syncretism conditions with standard deviation in brackets for each register, role (A = agent, P = patient) and word order condition. Means and 95% credible interval of posterior differences between ‘AVP’, a particular role and register and each corresponding combination of word order, role and register. For instance, for row ‘PAV’ and column ‘CDS_A’: ‘CDS_A’: *P*_AVP_(correct classification|register = CDS, role = agent) − *P*_PAV_(correct classification|register = CDS, role = agent). Accuracies vary across word orders with high accuracies in AVP, APV and VAP, mixed accuracies for PAV and VPA, and low accuracies for PVA.

Type	Role	CDS		ADS	
		Accuracy	Estimated difference	Accuracy	Estimated difference
AVP	A	0.98 (0.03)		0.98 (0.02)	
AVP	P	0.97 (0.02)		0.95 (0.04)	
APV	A	0.97 (0.02)	0.004 [0.003, 0.005]	0.95 (0.03)	0.0402 [0.039, 0.041]
APV	P	0.9 (0.03)	0.0666 [0.066, 0.068]	0.97 (0.02)	−0.014 [−0.015, −0.013]
PAV	A	0.87 (0.04)	0.115 [0.114, 0.116]	0.66 (0.15)	0.398 [0.397, 0.399]
PAV	P	0.72 (0.09)	0.1904 [0.190, 0.191]	0.64 (0.14)	0.343 [0.342, 0.345]
PVA	A	0.39 (0.23)	0.715 [0.71, 0.72]	0.18 (0.13)	0.869 [0.868, 0.8670]
PVA	P	0.46 (0.06)	0.502 [0.501, 0.504]	0.47 (0.13)	0.498 [0.496, 0.499]
VAP	A	0.82 (0.04)	0.159 [0.158, 0.160]	0.65 (0.2)	0.438 [0.436, 0.439]
VAP	P	0.98 (0.02)	−0.005 [−0.006, −0.004]	0.97 (0.02)	−0.0143 [−0.0149, −0.0136]
VPA	A	0.4 (0.23)	0.707 [0.706, 0.708]	0.19 (0.14)	0.8634 [0.863, 0.864]
VPA	P	0.91 (0.06)	0.097 [0.096, 0.098]	0.92 (0.05)	0.049 [0.048, 0.050]

**Figure 6.**
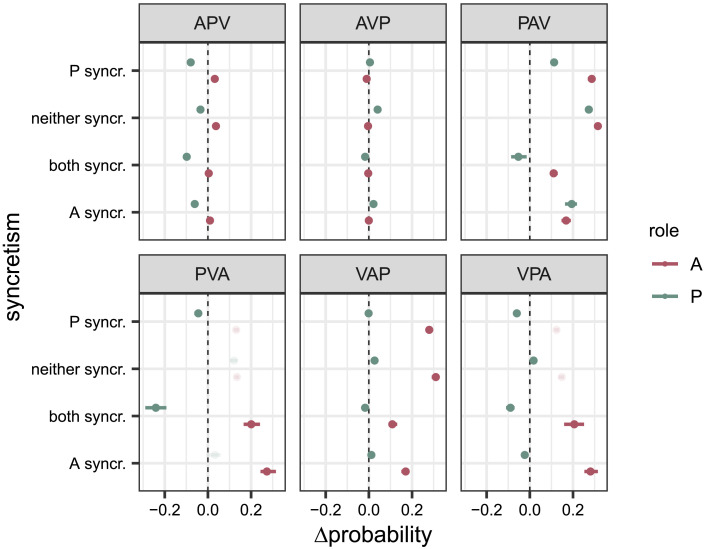
Pair-wise posterior differences between registers (CDS-ADS) for the Russian controlled dataset on the probability scale, (Δprobability), including mean and 95% credible interval, shown for both roles (A = agent, P = patient) and all conditions. Across most word orders and syncretism conditions, there is a positive effect of register for A, but only in some cases for P (e.g., ‘PAV’ except for ‘both syncretic’). A positive effect, i.e., mean pairwise difference larger than 0, indicates that models trained on CDS perform better at predicting semantic roles. For the word order ‘AVP’, no effect for register is found.

The estimated differences between registers also strongly vary across conditions (Figure 6). In the ‘APV’ word order, A arguments are classified more accurately based on CDS contextualised embeddings (*P*_both syncretic_(correct|CDS, A) − *P*_both syncretic_(correct|ADS, A) = 0.0041, [0.0037, 0.0044]; *P*_syncretic A_(correct|CDS, A) − *P*_syncretic A_(correct|ADS, A) = 0.01, [0.0091, 0.0099]; *P*_syncretic P_(correct|CDS, A) − *P*_syncretic P_(correct|ADS, A) = 0.031, [0.030, 0.032]; *P*_non-syncretic_(correct|CDS, A) − *P*_non-syncretic_(correct|ADS, A) = 0.037, [0.036, 0.038]), whereas for P arguments, the opposite is the case (*P*_bothsyncretic_(correct|CDS, P) − *P*_both syncretic_(correct|ADS, P) = −0.097, [−0.098, −0.095]; *P*_syncretic A_(correct|CDS, P) − *P*_syncretic A_(correct|ADS, P) = −0.062, [−0.063, −0.060]; *P*_syncretic P_(correct|CDS, P) − *P*_syncretic P_(correct|ADS, P) = −0.080, [−0.081, −0.079]; *P*_non-syncretic_(correct|CDS, P) − *P*_non-syncretic_(correct|ADS, P) = −0.035, [−0.036, −0.034]) In the ‘AVP’ word order, the effect of register for A is negligible with values below 0.01 (e.g., *P*_both syncretic_(correct|CDS, A) − *P*_both syncretic_(correct|ADS, A) = −0.0031, [−0.0033, −0.0029]). P arguments are classified more accurately based on CDS contextualised embeddings apart from when both arguments are syncretic (*P*_both syncretic_(correct|CDS, P) − *P*_both syncretic_(correct|ADS, P) = −0.0175, [−0.0181, −0.0168]; *P*_syncretic A_(correct|CDS, P) − *P*_syncretic A_(correct|ADS, P) = 0.021, [0.020, 0.022]; *P*_syncretic P_(correct|CDS, P) − *P*_syncretic P_(correct|ADS, P) = 0.0052, [0.0046, 0.0059]; *P*_non-syncretic_(correct|CDS, P) − *P*_non−syncretic_(correct|ADS, P) = 0.041, [0.040, 0.042]).

Regarding the ‘VAP’ word order, we find a higher probability for CDS with A (e.g., *P*_syncretic P_(correct|CDS, A) − *P*_syncretic P_(correct|ADS, A) = 0.279 [0.278, 0.281]), but mixed results with P (*P*_both syncretic_(correct|CDS, P) − *P*_both syncretic_(correct|ADS, P) = −0.0175, [−0.0181, −0.0168]; *P*_syncretic A_(correct|CDS, P) − *P*_syncretic A_(correct|ADS, P) = 0.012, [0.011, 0.013]; *P*_syncretic P_(correct|CDS, P) − *P*_syncretic P_(correct|ADS, P) = −0.0011, [−0.0016, −0.0005]; *P*_non−syncretic_(correct|CDS, P) − *P*_non-syncretic_(correct|ADS, P) = 0.0263, [0.0256, 0.0269]).

**Figure 7.**
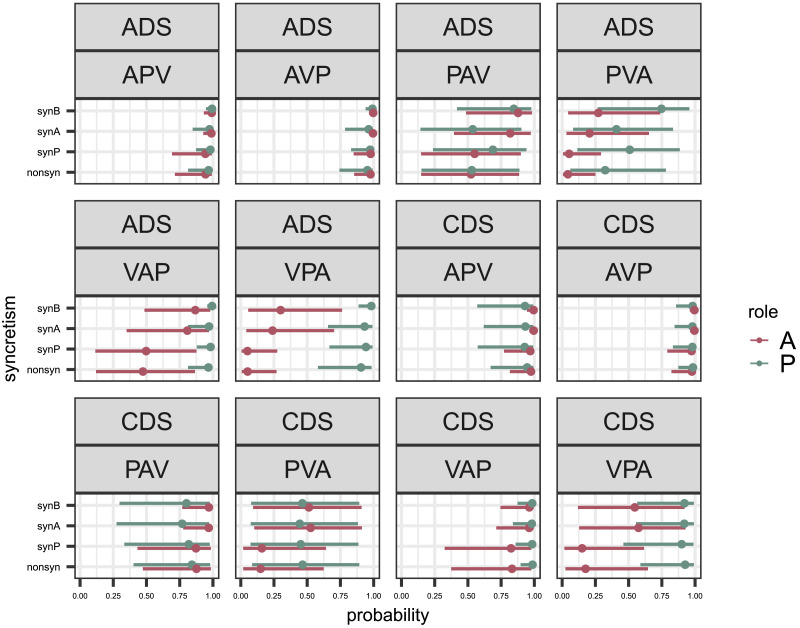
Posterior means and 95% credible interval for each syncretism condition grouped by register and role. Posterior means between syncretism conditions do not strongly differ with respect to a particular word order with means either very similar or credible intervals strongly overlapping.

With the ‘PAV’ word order, the contextualised embeddings trained on CDS perform better in predicting both roles across all conditions (e.g., *P*_syncretic P_(correct|CDS, A) − *P*_syncretic P_(correct|ADS, A) = 0.285, [0.283, 0.286] and e.g., *P*_syncretic P_(correct|CDS, P) − *P*_syncretic P_(correct|ADS, P) = 0.113, [0.111, 0.114]).

With respect to the utterances with word orders ‘PVA’ and ‘VPA’, considering only the ones for which an accuracy > 50% was reached, A arguments are better classified based on CDS (e.g., *P*_syncretic A, PVA_(correct|CDS, A) − *P*_syncretic A_(correct|ADS, A) = 0.274, [0.270, 0.277] and e.g., *P*_syncretic A, VPA_(correct|CDS, A) − *P*_syncretic A, VPA_(correct|ADS, A) = 0.282, [0.278, 0.285]) and P arguments on ADS (e.g., *P*_both syncretic, PVA_(correct|CDS, P) − *P*_both syncretic, PVA_(correct|ADS, P) = −0.239, [−0.244, −0.234] and e.g., *P*_both syncretic, VPA_(correct|CDS, P) − *P*_both syncretic, VPA_(correct|ADS, P) = −0.090, [−0.092, −0.088]).

### Discussion

In Study 2, we find that contextualised embeddings from both registers contain sufficient information to generalise across different structures. Overall, representations trained on CDS outperform those trained on ADS.

With regard to the English test set, the arguments were classified correctly to a high degree across semantic roles, conditions and registers. Only A in the passive condition was classified below chance based on contextualised embeddings trained on both registers. In spoken language, mentioning the A argument in a *by*-phrase in English is relatively rare (Gordon & Chafetz, 1990). The same pattern can be observed in our dataset with a low number of ‘PVA’ (0.522%, *n* = 11 in CDS and 1.1%, *n* = 26 in ADS) utterances and higher number of ‘PV’ sentences (1.471, *n* = 31 in CDS and 3.744, *n* = 89 in ADS) 2. Although both registers contain a high number of ‘AVP’ sentences in the training data, ‘PVA’ sentences, where P was either ‘fronted’ or the verb of P was in a ‘relative clause’, were still classified accurately. This suggests that the contextualised embeddings are flexible enough to generalise to less frequent structures, even though the classifier was exposed to only a few sentences from the less canonical conditions. The difference between registers shows that CDS yields a higher success of semantic role classification in the ‘relative clause’ condition. In the case of ‘fronted’ clauses, the same result only applies to P. Sentences in the ‘relative clause’ condition differ from the ones in the ‘fronted’ condition in two respects in our dataset. First, they are always introduced by ‘that’ and second, the matrix clause includes two additional words after the relative clause (‘relative clause’ *The stories **that** the friend knows are good* vs. ‘fronted’ *The stories the friend knows.*). These two additional cues may facilitate the classification of both roles more effectively in CDS than ADS, likely because similar structures are more frequently represented in the CDS dataset.

The fact that ‘AVP’ sentences are almost equally well predicted in both the prototypical and non-prototypical referent conditions suggests that the word order cue is robust enough to override potential mismatches in animacy-role prototypicality. Whether this strong reliance on word order would also apply to a child, however, is unclear. Supporting evidence for this may be the fact that English-speaking children rely on word order from a a young age to interpret sentence meaning, even in the absence of animacy cues (Bates et al., 1984; Chan et al., 2009; Gertner et al., 2006; Scott et al., 2018). The lack of a strong difference between registers indicates that as ceiling is approached, no additional boost from one of the two registers can be gained. However, in the majority of the remaining conditions (all apart from A in the ‘fronted’ condition), we see a benefit of CDS for semantic role classification.

Turning to the results for Russian, we observe consistently high accuracy for the most frequent word orders ‘AVP’ and ‘APV’ across semantic roles and registers. Thus, despite flexible word order in Russian, the tendency to interpret the first argument as A still applies, which is also expected based on the high number of A-initial sentences. In P-initial sentences, we observe a strong difference between the classification of P and A in ‘VPA’ sentences with a much higher accuracy for P. While ‘VP’ sentences are relatively frequent (ADS: 5.068, *n* = 63, CDS: 14.211, *n* = 108) in both CDS and ADS, ‘VPA’ sentences are rarely attested (CDS: 0.161, *n* = 2, ADS: 0.263, *n* = 2). In general, A-final sentences are extremely rare, explaining the low accuracy in ‘VPA’ sentences as well as ‘PVA’ sentences. However, the classification accuracy is relatively high for As in ‘PAV’ sentences where the P occurs in first position with the A argument occurring right after it before the verb.

For several word orders (‘APV’, ‘PVA’, ‘VPA’), we observe the tendency that A arguments are better classified using contextualised embeddings from CDS than ADS, while P arguments are better classified with embeddings from ADS. For the word order ‘AVP’, there is a slight advantage for ADS in classifying A arguments, while CDS shows a slight benefit for P arguments, except when both arguments have syncretic case marking. However, the effect sizes for A in the two most accurately classified word orders ‘AVP’ and ‘APV’, are relatively small (<0.01). This suggests that, as with English A-initial sentences, specific register provides little benefit when dealing with frequent, easily classified structures. Effect sizes for P are generally higher in these word orders with CDS favoring ‘AVP’ (unless both arguments are syncretic) and ADS favoring ‘APV’. Frequency alone does not explain these results, as the ‘AVP’ and ‘APV’ frequencies in each register do not align with the classification outcomes. For the ‘VAP’ word order, A arguments are generally more accurately classified with CDS, while classification results for P are mixed. In the ‘PAV’ word order, CDS consistently yields higher accuracy values for both roles across all syncretism conditions, except when both arguments are syncretic. The effect sizes are strong, ranging from 0.11 to 0.31. The higher proportion of ‘PAV’ sentences in CDS may explain this finding.

Overall, the positive effect sizes are larger (Δ*p*(*CDS* − *ADS*) > 0: mean = 0.135) than the negative effect sizes (Δ*p*(*CDS* − *ADS*) < 0: mean = −.05), indicating that when CDS predicts semantic roles more accurately, it does so with a stronger effect compared to ADS. Furthermore, across all conditions, the average mean of 0.07 (31 out of 48 of the conditions show positive effects for register) shows that overall contextualised embeddings trained on CDS perform better. In sum, A arguments, with a few exceptions, are predicted better based on CDS. P arguments are predicted better based on ADS whenever both arguments have syncretic case marking. In the absence of an unambiguous case marker, the only cue to semantic roles is subject-verb agreement. The majority of nominatives are singular in CDS while in ADS, a large part is also nominative plural. Thus, it is likely that information on agreement (i.e., plural vs. singular subject-verb agreement) is lacking in CDS. An additional reason may be that interactions with children more often involve animate referents than inanimates. Animate referents are more often marked by distinct forms than inanimates since some declension classes for inanimates have case syncretism between nominative and accusative singular. However, we do not find evidence for this in our descriptive analysis of case. CDS does not contain a higher proportion of non-syncretically case marked Ps than syncretically marked ones and the proportions are similar to the ones found in ADS (non-syncretic NOM and ACC case marking: CDS: 18.5% (*n* = 129); ADS: 15.2 % (*n* = 74), NOM and ACC singular syncretic case markings: CDS: 27.8% (*n* = 192); ADS: 21.4 % (*n* = 104)).

## GENERAL DISCUSSION

This study examined how CDS influences semantic role classification and learning, a fundamental step in acquiring syntactic rules from the input. Study 1 showed that semantic roles are more easily classified in utterances from CDS than ADS in Russian and partially also in English. Study 2 showed that in both languages, the statistical distributions of CDS enable more effective generalisation of semantic roles across a variety of structures. By using language models that learn from distributions in linguistic input, we showed that CDS provides a very informative source for learning semantic roles. The use of language models also allowed for comparing CDS and ADS directly. This methodology addressed limitations of previous studies that either described the characteristics of CDS in isolation, without a comparative baseline (e.g., Lester et al., 2022), or relied on linguistic labels not readily accessible to learners in the input (e.g., MacWhinney et al., 1984).

Role classification was easier in utterances from CDS than in ADS, possibly due to a more fixed word order in English CDS and lower variability of words and case markers in Russian CDS. This was supported by the classification of additional representations and the descriptive entropy analysis (see Results and Discussion). However, the lower variability in the expression of semantic roles in CDS does not hamper generalisability of semantic role knowledge. In fact, Study 2 indicated that embeddings learnt from CDS generalise roles to less frequent structures more successfully than those learnt from ADS (see Results and Discussion). In English, P-initial sentences were rare in both CDS and ADS. Despite this, just a few training examples of P-initial sentences were enough for the classifier to generalise semantic role knowledge to these less common structures, an effect that was stronger when using embeddings trained on CDS than on ADS. Indeed, CDS turns out to be particularly beneficial for structures that are less frequent in the input. With A-initial sentences, we do not find a difference between CDS and ADS in either language. Therefore, as performance reaches ceiling, there is less benefit to be gained from any specific input source. However, with less frequent, P-initial sentences, we find a higher predictability based on CDS in the majority of the conditions. Overall, sentences which are more frequent in CDS than ADS are also better classified based on CDS (e.g., ‘PAV’ word order in Russian). However, frequency is likely not the only factor driving the results of Study 2. For example, P-initial word orders are equally frequent in both registers in English.

We found a difference in effect size between the two languages in both studies. This result suggests that child-directed speech may be more strongly adapted to children in languages with a higher variety of word forms due to case marking and positions due to a more flexible word order for semantic roles. In Russian CDS, the number of and word forms that appear with a semantic role was lower than in Russian ADS (see Table 2). Interestingly, the position in which arguments occur was not more fixed in CDS than ADS. When speaking to children, Russian-speaking adults may use a lower variability of word forms to adapt to the knowledge of the child and to achieve successful communication. The low variability of word forms in Russian CDS may also be caused by the nature of the interactions with children and the topics talked about.

Low variability in the types of words occurring with the same word form may be caused by particular features of CDS previously described, namely the presence of repetition in the form of variation sets (Lester et al., 2022) or frequent frames (Mintz, 2003; Moran et al., 2018). Here, we demonstrate that these structures are indeed beneficial for understanding ‘who’ and ‘whom’ in natural utterances. Moreover, repetition does not seem to hamper the capability of generalising beyond the most frequent word orders. In Study 2, despite the prevalence of AVP sentences in English, the English BabyBERTa model correctly predicts PVA sentences, apart from A in the passive, which barely occurs in spoken language (Armon-Lotem et al., 2016). A similar tendency is also seen with Russian, however, the benefit of CDS is largely dependent on the particular word order and semantic role.

Our results also confirmed that the benefit of CDS in language development cannot simply be explained based on its prosodic characteristics that attract children’s attention (Gelderloos et al., 2020) or because of prosodic information that marks word, phrase, or utterance boundaries (Mandel et al., 1994; Nazzi et al., 2000; Soderstrom et al., 2003; Thiessen et al., 2005). An increased exposure to CDS enhances the child’s syntactic skills (Soderstrom, 2007), and here we show that this is also because of the structures found in CDS. We argue that both repetition and variation are key for syntactic development. Repetition has proven to be beneficial for inducing rules from the input as shown by a repetition in arguments leading e failure of predicting very rare structures correctly, such as the A argument in passive voice in English or PVA word order in Russian.

Importantly, we have shown that semantic roles can be inferred from the statistical properties of spoken language. The BabyBERTa model LMs in general are trained without any apriori knowledge of syntactic structures. Rather, linguistic knowledge emerges based on the exposure to raw linguistic input and the architectural features of the BabyBERTa model. This adds to the literature on the debate on whether the input provides enough information so that language-specific innate mechanisms are not a prerequisite to language acquisition (Pearl, 2022; Perfors et al., 2011). Certainly, the need for language-specific innate mechanisms cannot be disproved by a study like the present. Nevertheless, studying the input with language models reveals the extent to which a particular feature can be learnt based on linguistic input and how the learnability varies across different input types. Studying the input is crucial for arriving at a better understanding of how a child can learn any of the languages spoken today in very different cultural settings (Stoll & Lieven, 2013). Additionally, this line of research may reveal certain constraints of what can be learnt based on the input alone, which, as a consequence, may elucidate the role of discourse (Serratrice & Allen, 2015), prosodic (Grünloh et al., 2011) and extra-linguistic cues (Clerkin & Smith, 2022) that have not been taken into account in the present work. A blueprint to build models that take into account social context has recently been proposed by Tsuji et al. (2021). The combination of such a model with LMs, which can reveal what is learnable based on the input, is arguably a fruitful avenue for future research. Deploying LMs as tools to analyse the linguistic input has been predominantly done in studying English (e.g., You et al., 2021) and only few on other languages such as Korean (Shin & Mun, 2023). We acknowledge that the training data sizes for our BabyBERTa models are considerably smaller than they usually are for transformed-based models and that this could potentially strengthen or weaken effects found between registers. Nevertheless, our results show that even with relatively small training datasets, models can acquire (some) linguistic knowledge and exhibit clear differences across different types of training inputs. These findings underscore the potential for future research on languages where only limited data is available, paving the way for broader, cross-linguistic insights into language acquisition.

## ACKNOWLEDGMENTS

We are grateful to the two anonymous reviewers and the editor, Ted Gibson, for their valuable feedback. We also thank members of the ISLE institute at the University of Zurich as well as audiences at CogSci 2024 and IASCL 2024 for helpful insights, discussion, and feedback.

## FUNDING INFORMATION

NCCR Evolving Language, Swiss National Science Foundation Agreement No. 51NF40_180888.

## AUTHOR CONTRIBUTIONS

EH: Conceptualization; Data curation; Formal analysis; Investigation; Methodology; Software; Visualization; Writing – original draft; Writing – review & editing. BB: Conceptualization; Funding acquisition; Supervision; Writing – review & editing. SS: Conceptualization; Funding acquisition; Supervision; Writing – review & editing.

## DATA AVAILABILITY STATEMENT

The English data is available on the OSF repository (https://osf.io/4tevz/?view_only=9c5af7e3cd9e493a886808e7c47fc206). The Russian data can be shared upon request through the ACQDIV Corpus (https://www.isle.uzh.ch/en/ACQDIV/resources.html).

## Notes

^1^ See Supplementary Material, directory ‘guidelines’.^2^ See Huebner et al. (2021) for the full list of alterations.^3^ We performed a sensitivity analysis to ensure that the differences found between English and Russian are not due to the fact that the Russian ADS corpus contains subtitle data besides naturalistic conversations (see S2 in file ‘supplementary_material_additional_analysis’). We find no evidence that differences between the languages are attributable to the different register in the ADS datasets.
